# Progression rate of diverticular disease and associated risk factors: results from 5-year longitudinal prospective nationwide diverticular disease registry (REMAD)

**DOI:** 10.1007/s11739-026-04283-4

**Published:** 2026-02-26

**Authors:** Marilia Carabotti, Caterina Sbarigia, Giuseppe Campagna, Rosario Cuomo, Fabio Pace, Camilla Ritieni, Giovanni Barbara, Bruno Annibale

**Affiliations:** 1 Department of Medical-Surgical Sciences and Translational Medicine, Sapienza University of Rome, Via Di Grottarossa 1035-1039, 00189 Rome, Italy; 2 Gastroenterology and Endoscopy Unit, “Sant’Anna E San Sebastiano” Hospital, Caserta, Italy; 3 Head Complex Operating Unit of Gastroenterology, “Bolognini” Hospital, Seriate, Italy; 4 Department of Medical and Surgical Sciences, University of Bologna, Bologna, Italy

**Keywords:** Diverticular disease, Acute diverticulitis, Symptomatic uncomplicated diverticular disease, Risk factors, Natural history

## Abstract

Natural history of diverticular disease (DD) through its different subgroups (diverticulosis, symptomatic uncomplicated diverticular disease (SUDD) previous diverticulitis (PD)) is poorly defined. Few studies, mainly retrospective, have analysed its rate of progression and risk factors associated. We assessed the potential progression rate of different spectrum of DD and associated risk factors. Data from the REMAD Registry, a 5-year Italian longitudinal prospective multicentre study, were analysed. A total of 1217 patients were included that were divided into three subgroups (diverticulosis, SUDD and PD) and followed up every six months for 5 years. Amongst patients with colonic diverticulosis, about 7% progressed to SUDD with an average follow-up period of 18.3 (SD ± 16.1) months, whilst 1% had an acute episode of diverticulitis with a mean time to progression of 23.7 (SD ± 15.0) months. Amongst SUDD, 2.7% progressed to acute diverticulitis with a mean follow-up time of 29.2 (SD ± 17.1) months. Almost 20% of patients with PD had a first recurrence at a mean follow-up of 20.1 (SD ± 14.2) months, whilst 14.3% had a second recurrence at a mean follow-up of 15.6 (SD ± 7.0) months. Gender, diet (low fibre intake and meat consumption) being overweight, gastrointestinal tract medications and antiplatelet use are risk factors involved in DD progression. This longitudinal prospective study demonstrates a low progression rate in diverticulosis and SUDD, whereas diverticulitis recurrences are a frequent occurrence, representing the major clinical burden in DD. These results suggest that DD can progress, highlighting the need to understand how to prevent it from developing into a symptomatic and/or complicated form.

**Supplementary Information:**

The online version contains supplementary material available at 10.1007/s11739-026-04283-4.

## Introduction

Diverticular disease (DD) is a significant health issue in Western countries, primarily due to its high prevalence, which increases with age, affecting nearly 75% of individuals over 80 years old [1–3].

Whilst most people with colonic diverticula will remain asymptomatic throughout their lifetime (a condition known as diverticulosis), a proportion of them will develop either recurrent lower abdominal symptoms without signs of inflammation, a condition known as symptomatic uncomplicated diverticular disease (SUDD), or complications, such as acute diverticulitis [4, 5].

The progression of DD has been difficult to investigate over time due to several limitations, such as a lack of well-designed, long-term, prospective studies. There are also uncertainties relating to the different clinical scenarios of DD and the diagnostic methods used to categorise each disease subtype, including SUDD definition and its diagnostic criteria [6]. Therefore, a few studies have analysed the progression from diverticulosis to SUDD [7] or the progression from SUDD to acute diverticulitis [8–11].

Conversely, the incidence of acute diverticulitis in patients with diverticulosis has historically been estimated at 30% [12], but these data are based on older literature and subsequent retrospective studies have revised this figure downwards [13–16].

Finally, the natural history of patients with a previous episode of diverticulitis (PD) and the risk of recurrences have been investigated in several studies, both retrospective and prospective. Recent evidence suggests that recurrences occur in up to 36% of patients with a higher incidence in the first 12 months after the initial episode of acute diverticulitis [17–20].

Furthermore, the risk factors involved in the progression of DD have mainly been analysed in the context of acute diverticulitis [4] sometimes with conflicting results, whilst literature data concerning the progression of SUDD are scarce.

The wide spectrum of DD and its potential progression to various clinical scenarios (e.g. diverticulosis progressing to either SUDD or acute diverticulitis; SUDD progressing to acute diverticulitis and PD progressing to recurrences) poses challenges to assess its natural history.

This longitudinal prospective study aimed to evaluate DD progression rate and its associated risk factors. Specifically, we aimed to evaluate

1. The incidence of SUDD and acute diverticulitis amongst patients with diverticulosis
2. The incidence of acute diverticulitis amongst patients with SUDD
3. The recurrence of acute diverticulitis in patients with previous diverticulitis.

## Materials and methods

### Study design and population

Data were collected from the Diverticular Disease Registry (REMAD), which was promoted by the Italian Study Group on Diverticular Disease (GrIMAD). This 5-year longitudinal prospective observational multicentre study (April 2015–April 2020) was designed to assess the clinical characteristics and progression of diverticular disease.

Materials and methods have been reported extensively in previous papers [21–24]. Briefly, 47 Italian centres consecutively enrolled a total of 1217 DD patients over 2-month recruitment period (from April 30, 2015 to June 30, 2015). Inclusion criteria were ability to sign the informed consent, age ≥18 years and colonic diverticula confirmed by endoscopic or imaging examination. Failure to sign the informed consent form and inability to adhere to the study procedures were the exclusion criteria.

### DD classification and data collection

At baseline, patients were divided into three subgroups [21–24]: (a) Diverticulosis: presence of colonic diverticula in the absence of lower abdominal symptoms; (b) SUDD: presence of colonic diverticula and recurrent abdominal pain, mainly in the lower abdominal quadrants, at least once a week, present for at least six months and/or changes in bowel habits [25, 26] and (c) Previous diverticulitis (PD): patients with at least one previous episode of acute diverticulitis, complicated or not.

During follow-up, a diagnosis of acute diverticulitis, either first episode or a recurrence, was made based on clinical evaluation in the presence of abdominal pain lasting more than 24 hours and at least three of the following criteria: 1) fever; 2) need for bed rest; 3) need for medical consultation; 4) use of antibiotic therapy and 5) need for hospitalisation.

The study protocol involved patients’ assessment every six months, alternating between telephone and face-to-face visits, for a total of 11 visits per patient over five years of follow-up. At baseline, the following data were collected: demographical and personal data (body mass index [BMI] and first-degree family history of DD), lifestyle factors (cigarette smoking, diet and coffee consumption), concomitant medications (NSAIDs, antiplatelets and statins) including those commonly used for DD (rifaximin, mesalazine, prebiotics and probiotics). At each visit, patients’ clinical status was assessed using structured questionnaires as previously reported [24, 25, 27].

The protocol was approved by the coordinating centre University Federico II, Naples, on 24 September 2014 (approval identification no: 161/14) and by independent ethics committees at each centre. The study was carried out according to the Declaration of Helsinki and the principles of good clinical practice. Written informed consent was provided by all patients. The study was registered on a public registry (ClinicalTrial.gov no: NCT03325829).

### Statistical analysis

Continuous variables were presented as mean ± standard deviation (SD) and 95%CI (Confidence Interval), and categorical variables were expressed as absolute frequency and percentage, *n* (%).

Comparison of the continuous variables was performed by Generalized Linear Mixed Model (GLIMMIX) with Gaussian distribution, and post hoc analysis was performed by Tukey method; whilst the association between categorical variables and groups was evaluated by *X*^2^ or Freeman–Halton–Fisher test. Results were obtained using multinomial regression with cumulative logit function. In each table, the reference levels were SUDD, diverticulitis and recurrences. Multinomial regression has allowed to identify potential factors associated with DD progression: age, gender, BMI, first-degree family history of DD, low-fibre diet (less than 21 portions of fruit, vegetables and/or whole foods per week), high meat consumption (more than 3 portions per week), active smoking, coffee use (low: ≤2/die or high: ≥3/die), medical treatments with NSAIDs (regular: at least once a week), antiplatelets and statins, rifaximin (at least one cycle of 7–10 days per month in the last year), mesalazine, probiotics and prebiotics (at least one cycle of 10 days per month in the last year).

The Sankey diagram was used to show the change in data flow over the follow-up where the width of the flows is proportional to the state of disease progression.

Kaplan–Meier curves were plotted to show the progression of the disease, i.e. the different stages of progression (diverticulosis, SUDD, PD and acute diverticulitis recurrences).

Statistical analysis was performed using SAS version 9.4 TS Level 1 M8 and JMP PRO version 17 (SAS Institute, Cary, NC, USA). A *p* value < 0.05 was considered statistically significant.

## Results

### Baseline characteristics

Of the 1217 patients enrolled in the REMAD registry, 705 (57.9%) were diagnosed with diverticulosis, 300 (24.7%) with SUDD and the remaining 212 (17.4%) with PD.

Table S1 provides a detailed overview of the differences in baseline demographical and clinical characteristics, lifestyle and dietary factors and medications between the subgroups. In brief, significant differences were observed between the subgroups (diverticulosis, SUDD and PD) at baseline in terms of gender (*p*<0.0001), age (*p*=0.003), first-degree family history of DD (*p*=0.007) and medications [rifaximin (*p*<0.0001), mesalazine (p<0.0001) and probiotics (*p*<0.0001)].

### Adherence to follow-up

Overall, Adherence was 80.7%, 65.0%, 52.0%, 41.4% and 30.5% at one, two, three, four and five years, respectively. There was a significant difference in the length of follow-up between the subgroups [diverticulosis: 35.9 (95% CI: 34.2 to 37.5) months; SUDD: 38.6 (95% CI: 36.1 to 41.1) months and PD: 39.6 (95% CI: 35.4 to 41.9) months; *p*=0.03], which was significantly higher only in the PD subgroup compared to the diverticulosis subgroup (*p*=0.01).

### DD Progression rate

**Fig. 1:**
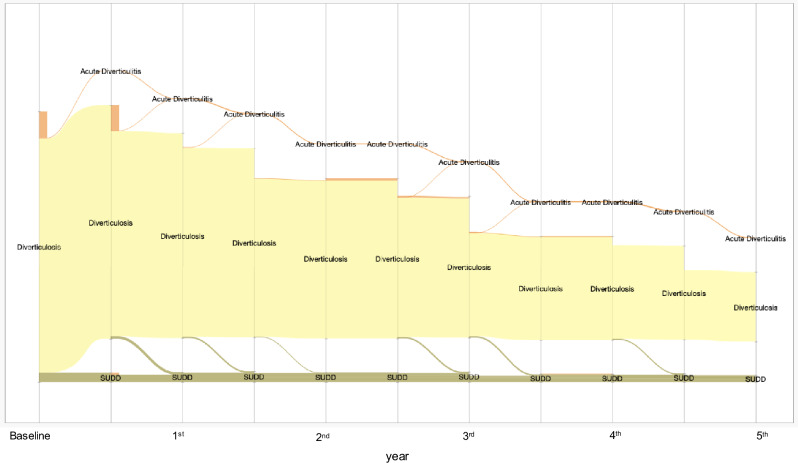
Sankey diagram reporting the progression rate of patients with baseline diverticulosis Legend: The main flow is represented by patients with baseline diverticulosis who progressed to SUDD or acute diverticulitis during the follow-up

**Fig. 2:**
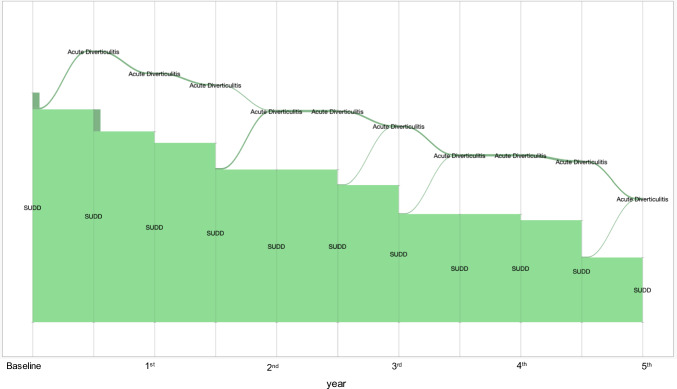
Sankey diagram reporting the progression rate of patients with baseline symptomatic uncomplicated diverticular disease (SUDD) Legend: The main flow is represented by patients with baseline symptomatic uncomplicated diverticular disease (SUDD) who progressed to acute diverticulitis during the follow-up

**Fig. 3:**
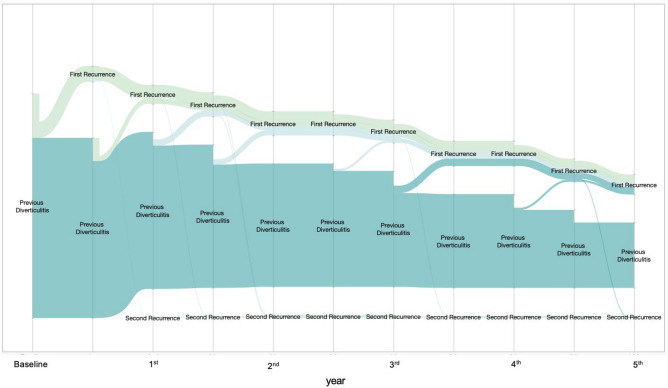
Sankey diagram reporting the progression rate of patients with baseline previous diverticulitis (PD) Legend: The main flow is represented by patients with baseline previous diverticulitis (PD) who progressed to a first and second recurrence during the follow-up

Figures 1, 2 and 3 show the rate and time to progression from baseline for diverticulosis, SUDD and PD, respectively.

In brief, 7.2% of patients in the diverticulosis subgroup progressed to SUDD, with a mean time to progression of 18.3 (±16.1) months. Analysing incidence through Kaplan–Meier analysis, 51 patients with baseline diverticulosis developed SUDD (7.2%; 2.4 per 100 person-year) (Figure S1). Meanwhile, 1.1% progressed to diverticulitis, with a mean time to progression of 23.7 (±15.0) months, with no recurrences during follow-up (Figure 1). Kaplan–Meier analysis show that 8 patients with baseline diverticulosis developed diverticulitis (1.1%; 0.4 per 100 person-year) (Fig. S2).

Amongst the SUDD subgroup, 2.7% experienced a first episode of diverticulitis at an average follow-up period of 29.2 (±17.1) months (Fig. 2). Eight patients with baseline SUDD developed diverticulitis (2.7%; 0.9 per 100 person-year) (Fig. S3). No significant difference in terms of progression to acute diverticulitis was observed between diverticulosis and SUDD patients (1.1% vs 2.7%, *p*=0.08). Amongst new acute diverticulitis diagnoses, there were no recurrences during the five-year follow-up period.

Almost 20% of patients with PD experienced a first recurrence at a mean follow-up time of 20.1 (± 14.25) months, whilst 14.3% experienced a second recurrence at a mean follow-up time of 15.6 (± 7.0) months (Figure 3). Forty-eight patients with baseline PD developed a recurrent diverticulitis (22.6%; 7.7 per 100 person-year) (Figure S4).

### Factors associated with DD progression

#### Progression from diverticulosis to SUDD

In patients with diverticulosis, the significant risk factors for progression to SUDD were male gender, regardless of age grouping > or < 60 years (*p*=0.03 and *p*<0.0001, respectively), being overweight (*p*=0.03) and high meat consumption (*p*=0.01) and not taking medications related to the gastrointestinal tract [rifaximin (*p*<0.0001), mesalazine (<0.0001), prebiotics (*p*=0.002) and probiotics (*p*=0.003)] (Table 1).

**Table 1:** Risk factors for progression from diverticulosis to symptomatic uncomplicated diverticular disease (SUDD)

Parameters	OR (95%CI)	*p* value
Age classes*Sex		
< 60 years* Female vs. Male*	0.77 (0.60 to 0.98)	**0.03**
≥ 60 years* Female vs. Male*	0.78 (0.70 to 0.86)	** < 0.0001**
BMI classes (kg/m^2^)		
< *25 vs.* ≥ *25*	0.87 (0.77 to 0.99)	**0.03**
First-degree family history of DD		
*No vs. Yes*	1.14 (0.99 to 1.32)	0.08
Diet		
*Low vs. High Fibre*	1.16 (0.99 to 1.37)	0.07
Meat		
*Low vs. High*	0.86 (0.76 to 0.97)	**0.01**
Smoking		
*No vs. Yes*	0.95 (0.81 to 1.12)	0.54
Coffee intake		
*Low vs. High*	0.99 (0.87 to 1.23)	0.88
NSAIDs		
*No vs. Yes*	1.14 (0.97 to 1.34)	0.12
NSAIDs Frequency		
*Regular vs. Other*	0.72 (0.52 to 1.00)	0.052
Antiplatelets		
*Single and/or double vs. Not used*	0.95 (0.81 to 1.12)	0.54
Statins		
*No vs. Yes*	1.04 (0.89 to 1.22)	0.64
Rifaximin		
*No vs. Yes*	3.00 (2.57 to 3.49)	** < 0.0001**
Mesalazine		
*No vs. Yes*	3.08 (2.16 to 4.40)	** < 0.0001**
Prebiotics		
*No vs. Yes*	2.52 (1.40 to 4.55)	**0.002**
Probiotics		
*No vs. Yes*	1.74 (1.20 to 2.52)	**0.003**

*Legend: BMI* body mass index; *DD* diverticular disease; *NSAIDs* non-steroidal antinflammatory drugs.* The p value in bold is statistically significant (p value <0.05).*

#### Progression from diverticulosis to acute diverticulitis

Risk factors for progression from diverticulosis to acute diverticulitis were female gender patients aged ≥60 years (*p*=0.002), being overweight (*p*<0.0001), absence of a first-degree family history of DD (*p*=0.001) and low-fibre diet (p=0.006) and no use of rifaximin (*p*<0.0001), mesalazine (*p*<0.0001) or probiotics (*p*=0.001) (Table 2).

**Table 2:** Risk factors for progression from diverticulosis to diverticulitis

Parameters	OR (95%CI)	*p* value
Age classes*Sex		
< 60 years* Female vs. Male*	0.97 (0.74 to 1.27)	0.83
≥ 60 years* Female vs. Male*	1.22 (1.07 to 1.38)	**0.002**
BMI classes (kg/m^2^)		
< *25 vs.* ≥ *25*	0.71 (0.61 to 0.83)	** < 0.0001**
First-degree family history of DD		
*No vs. Yes*	1.32 (1.11 to 1.57)	**0.001**
Diet		
*Low vs. High Fibre*	1.31 (1.08 to 1.60)	**0.006**
Meat		
*Low vs. High*	1.14 (0.98 to 1.33)	0.09
Smoking		
*No vs. Yes*	1.04 (0.86 to 1.26)	0.69
Coffee intake		
*Low vs. High*	0.96 (0.81 to 1.13)	0.59
NSAIDs		
*No vs. Yes*	0.94 (0.77 to 1.15)	0.55
NSAIDs frequency		
*Regular vs. Other*	0.94 (0.59 to 1.49)	0.78
Antiplatelets		
*Single and/or double vs. Not used*	1.21 (0.97 to 1.50)	0.09
Statins		
*No vs. Yes*	0.95 (0.77 to 1.17)	0.63
Rifaximin		
*No vs. Yes*	4.90 (4.11 to 5.83)	** < 0.0001**
Mesalazine		
*No vs. Yes*	4.81 (3.34 to 6.93)	** < 0.0001**
Prebiotics		
*No vs. Yes*	1.58 (0.76 to 3.31)	0.22
Probiotics		
*No vs. Yes*	1.98 (1.31 to 2.99)	**0.001**

*Legend: BMI* body mass index; *DD* diverticular disease; *NSAIDs* non-steroidal antinflammatory drugs. *The p value in bold is statistically significant (p value <0.05)*

#### Progression from SUDD to diverticulitis

Risk factors for progression from SUDD to acute diverticulitis were female gender in patients aged ≥60 years (*p*<0.0001), being overweight (p=0.01), low meat consumption (*p*=0.0005) taking antiplatelets (*p*=0.04) and not taking any rifaximin or mesalazine (*p*<0.0001 and *p*=0.005, respectively) (Table 3).

**Table 3:** Risk factors for progression from symptomatic uncomplicated diverticular disease (SUDD) to diverticulitis

Parameter	OR (95%CI)	*p* value
Age classes*Sex		
< 60 years* Female vs. Male*	1.27 (0.96 to 1.68)	0.10
≥ 60 years* Female vs. Male*	1.56 (1.37 to 1.78)	** < 0.0001**
BMI classes (kg/m^2^)		
< *25 vs.* ≥ *25*	0.81 (0.69 to 0.96)	**0.01**
First-degree family history of DD		
*No vs. Yes*	1.16 (0.97 to 1.39)	0.11
Diet		
*Low vs. High Fibre*	1.13 (0.92 to 1.39)	0.24
Meat		
*Low vs. High*	1.33 (1.13 to 1.56)	**0.0005**
Smoking		
*No vs. Yes*	1.10 (0.89 to 1.35)	0.39
Coffee intake		
*Low vs. High*	0.97 (0.81 to 1.15)	0.71
NSAIDs		
*No vs. Yes*	0.83 (0.67 to 1.03)	0.08
NSAIDs frequency		
*Regular vs. Other*	1.30 (0.82 to 2.06)	0.26
Antiplatelets		
*Single and/or double vs. Not used*	1.27 (1.02 to 1.59)	**0.04**
Statins		
*No vs. Yes*	0.92 (0.74 to 1.14)	0.42
Rifaximin		
*No vs. Yes*	1.63 (1.38 to 1.94)	** < 0.0001**
Mesalazine		
*No vs. Yes*	1.56 (1.15 to 2.12)	**0.005**
Prebiotics		
*No vs. Yes*	0.63 (0.32 to 1.23)	0.17
Probiotics		
*No vs. Yes*	1.14 (0.78 to 1.66)	0.51

*Legend: BMI* body mass index; *DD* diverticular disease; *NSAIDs* non-steroidal antinflammatory drugs. * The p value in bold is statistically significant (p value <0.05)*

#### Progression from PD to recurrences

In patients with previous diverticulitis, recurrences were significantly associated with male gender regardless of grouping > or < 60 years (*p*=0.01 and *p*<0.0001, respectively), and absence of a first-degree family history of DD (*p*=0.006) (Table 4).

**Table 4:** Risk factors for diverticulitis recurrences

Parameters	OR (95%CI)	*p* value
Age classes*Sex		
< 60 years *Female vs. Male*	0.47 (0.27 to 0.84)	**0.01**
≥ 60 years *Female vs. Male*	0.42 (0.31 to 0.56)	** < 0.0001**
BMI classes (kg/m^2^)		
< *25 vs.* ≥ *25*	1.16 (0.81 to 1.66)	0.42
First-degree family history of DD		
*No vs. Yes*	1.66 (1.16 to 2.38)	**0.006**
Diet		
*Low vs High fibre*	0.65 (0.40 to 1.06)	0.08
Meat		
*Low vs. High*	0.95 (0.67 to 1.35)	0.78
Smoking		
*No vs. Yes*	1.39 (0.91 to 2.13)	0.13
Coffee intake		
*Low vs. High*	0.86 (0.58 to 1.27)	0.45
NSAIDs		
*No vs. Yes*	1.08 (0.68 to 1.73)	0.74
NSAIDs frequency		
*Regular vs. Other*	2.68 (0.60 to 11.98)	0.21
Antiplatelets		
*Single and/or double vs. Not used*	0.63 (0.39 to 1.03)	0.06
Statins		
*No vs. Yes*	1.15 (0.71 to 1.84)	0.58
Rifaximin		
*No vs. Yes*	0.64 (0.43 to 1.93)	0.53
Mesalazine		
*No vs. Yes*	1.71 (0.97 to 2.99)	0.06
Prebiotics		
*No vs. Yes*	0.57 (0.07 to 4.50)	0.59
Probiotics		
*No vs. Yes*	0.67 (0.28 to 1.65)	0.38

*Legend: BMI body mass index; DD diverticular disease; NSAIDs non-steroidal antinflammatory drug. The p value in bold is statistically significant (p value <0.05)*

## Discussion

To the best of our knowledge, this is the first cohort study to prospectively evaluate the natural history of DD considering all its different subgroups (diverticulosis, SUDD and PD) and associated risk factors.

The first interesting finding is that in patients with colonic diverticulosis, progression to SUDD is approximately 7%. Only one prospective study has investigated the progression from colonic diverticulosis to SUDD, showing an incidence of 1% during a follow-up period of nearly 7 years [7], although another retrospective study reported that almost 25% of patients with colonic diverticulosis develop SUDD-related symptoms during an average follow-up of about 13 years [11]. Compared to our results, the different rate of progression could be explained by the different criteria used to diagnose SUDD: in fact, both Peery et al. and Tursi et al. defined SUDD as the presence of left-sided abdominal pain lasting at least 24 consecutive hours [7, 11], thus excluding short-lasting (<24 hours) abdominal pain, whilst we used less restrictive to define SUDD, in terms of both the site of the abdominal pain (all quadrants) and its duration (> and < 24 hours). Furthermore, Peery et al. only collected their data by telephone interview [7], whereas we used both telephone and face-to-face interviews. The latter are known to be more effective for data collection than telephone interviews, as they allow direct interaction with patients and reduce recall bias [28]. Moreover, the definition of SUDD itself is debated and there is no shared consensus on diagnostic criteria [6]. Nevertheless, abdominal symptoms do occur in patients with diverticulosis and are significant from a clinical perspective.

Another finding is the progression from SUDD to acute diverticulitis that occur in nearly 3% of patients, over an average period of 29 months. According to the available evidence, the progression rate is estimated to be between 1.7 and 10.4%. However, this wide range is again due to the different criteria used for diagnosing SUDD, as well as the varying lengths of follow-up (from five to 13 years) and study design [8, 9, 11].

Another interesting point is that the progression from colonic diverticulosis directly to acute diverticulitis is essentially an infrequent event. In fact, according to our data, it occurs in approximately 1.1% of patients with diverticulosis, within an average period of 24 months. Previous studies have reported an incidence of acute diverticulitis ranging from 2.8 to 6.9% [13–16], but these were all retrospective. Interestingly, some of these studies used stricter criteria to define diverticulitis events (e.g. assessment made by imaging and/or surgery), and when these stricter criteria were applied, the incidence of diverticulitis was around 1% [13, 14], which is consistent with our findings.

These results confirm that DD is a condition which can progress, raising the question of how to prevent its development into a symptomatic and/or complicated form.

We showed that nearly one-fifth of PD cases had a recurrence after an average of 20 months, whilst a second recurrence occurred in 15% of patients, after an average period of 16 months. These findings confirm that recurrences after the first acute episode are the major clinical challenge in DD, as has been shown extensively [5, 19, 20, 29].

In terms of risk factors for the progression of DD, we showed that female gender is a risk factor for acute diverticulitis in patients over 60 years of age, as previously reported in epidemiological studies [4, 30–33]. However, we also found that male gender plays a role in the progression of DD, being a risk factor for the development of SUDD from diverticulosis (*p*=0.03 and *p*<0.0001) and for AD recurrence (*p*=0.01 and *p*<0.0001), irrespective of age. Therefore, the future agenda should consider the potential role of gender and all its different implications in DD progression.

In our cohort, being overweight (BMI ≥25 kg/m^2^) was associated with an increased risk of developing both SUDD (*p*=0.03) and acute diverticulitis (from diverticulosis: *p*=0.002, from SUDD: p=0.01) confirming its negative role in DD [34]. The biological role of obesity in DD is not well established, although several hypotheses have been tested, including the pro-inflammatory effect of the cytokines released from the adipose tissue [35, 36] as well as changes in the gut microbiota associated with obesity [26].

Surprisingly, no family history of DD significantly increased the risk of diverticulitis, both for the first episode (*p*=0.001) and recurrences (*p*=0.006). The genetic contribution to DD has been suggested by several studies [37, 38]; however, as DD is often asymptomatic, it is possible that people may not be aware that they have diverticula, thus underestimating the frequency of a known family history of DD.

In terms of diet, we showed that high meat consumption was a risk factor for progression of diverticulosis to SUDD (*p*=0.01), whereas low meat consumption was a risk factor for progression of SUDD to diverticulitis (*p*=0.0005). However, according to the literature, only red meat consumption is an established risk factor for DD complications [39, 40]. This discrepancy may be explained by the fact that we did not distinguish between red and white meat; therefore, we cannot draw any specific conclusions about their respective roles in the progression of DD. Furthermore, we confirmed that a low-fibre diet is a risk factor for diverticulitis (*p*=0.006), as previously reported [39, 41, 42].

In addition, antiplatelet therapy has emerged as a factor that promotes progression from SUDD to diverticulitis. Whilst some data suggest a role in the onset of diverticular bleeding [43], less evidence is available regarding the risk of diverticulitis associated with the use of these drugs. This aspect that warrants further investigation with ad hoc studies.

In terms of medications commonly used in DD, we found that patients who were not receiving rifaximin, mesalazine, or probiotics were at increased risk of disease progression, either to SUDD or acute diverticulitis. Although these findings suggest a potentially protective role, they should be interpreted with caution, as they derive from an observational, non-interventional study in which treatment regimens may not have been homogeneous and treatment adherence was not assessed. Although no solid evidence is currently available regarding the role of these medications in preventing acute episodes, either in primary or secondary prophylaxis [44], they are widely prescribed in clinical practice in Italy [22]. The lack of robust evidence regarding therapeutic options for symptom improvement in patients with SUDD, as well as for the primary and secondary prevention of acute diverticulitis, remains an important unmet need in the management of DD, and the cost-effectiveness of such therapeutic strategies still needs to be established in dedicated studies.

This study has some limitations, the most important being that almost 60% of patients were lost to follow-up during the five-year period, particularly within the diverticulosis and SUDD groups. This may be because patients with a history of acute diverticulitis tend to be more compliant than patients with diverticulosis or SUDD, as they have already experienced a complication. Patients lost to follow-up may reduce the consistency of the results, but this is a common selection bias in cohort studies [45]. Secondly, diagnosis of acute diverticulitis (whether first episode or recurrence) during follow-up was based on clinical criteria, so we cannot exclude an overestimation of our results. Indeed, in the present study, SUDD was defined according to the best available clinical criteria when the study protocol was developed. Notably, no shared diagnostic criteria were available at that time, and this remains the case to this day. Currently, the diagnosis of SUDD remains a matter of debate as there is no universally accepted consensus [6]. The absence of shared diagnostic criteria and validated biomarkers represents a significant unmet need in the field of diverticular disease.

Nevertheless, this is the first study to prospectively evaluate the progression of different DD subgroups through structured follow-up visits, highlighting that specific factors such as gender, age, overweight, diet and drugs use may influence disease progression.

This longitudinal prospective study demonstrates a low progression rate in diverticulosis and SUDD, whereas diverticulitis recurrences are a frequent occurrence, representing the major clinical burden in DD. These results suggest that DD can progress, highlighting the need to understand how to prevent it from developing into a symptomatic and/or complicated form.

## Supplementary Information

Below is the link to the electronic supplementary material.

Supplementary file1 (DOCX 20 KB)

Supplementary file2 (PDF 52 KB)

Supplementary file3 (PDF 54 KB)

Supplementary file4 (PDF 51 KB)

Supplementary file5 (PDF 51 KB)

## Data Availability

Data supporting this study are available from the corresponding author upon reasonable request.
