# Supplementary material for: Progression rate of diverticular disease and associated risk factors: results from 5-year longitudinal prospective nationwide diverticular disease registry (REMAD)
Source: Intern Emerg Med. 2026 Feb 26;21(4):1215–25. doi: 10.1007/s11739-026-04283-4 (PMC13263300; doi:10.1007/s11739-026-04283-4)
Supplement: Supplementary file 1 — Supplementary file1 (DOCX 20 KB) [file 11739_2026_4283_MOESM1_ESM.docx]

Table S1. Clinical features, lifestyle factors and drug assumption in diverticulosis, symptomatic uncomplicated diverticular disease and previous diverticulitis patients at baseline.

|  | **Diverticulosis** n=705 | **SUDD** n=300 | **Previous**  **diverticulitis** n=212 | p value |
| --- | --- | --- | --- | --- |
| **Demographic and**  **clinical characteristics** |  |  |  |  |
| Gender |  |  |  | **<0.0001** |
| *Female, n (%)* | 277 (39.29) | 176 (58.67) | 109 (51.42) |  |
| *Male, n (%)* | 428 (60.71) | 124 (41.33) | 103 (48.58) |  |
| Age (years), mean±SD; (95%CI) | 66.68±9.43; (65.99 to 67.38) | 66.26±9.55; (65.17 to 67.35) | 64.01±11.59; (62.45 to 65.58) | **0.003** |
| Age classes |  |  |  | **<0.0001** |
| *<60 years, n (%)* | 148 (20.99) | 74 (24.67) | 77 (36.32) |  |
| *≥60 years, n (%)* | 557 (79.01) | 226 (75.33) | 135 (63.68) |  |
| Age classes*Sex |  |  |  |  |
| <60 years |  |  |  | 0.68 |
| *Female, n (%)* | 53 (35.81) | 32 (43.24) | 30 (39.47) |  |
| *Male, n (%)* | 95 (64.19) | 42 (56.76) | 46 (60.53) |  |
| ≥60 years, n (%) |  |  |  | **0.029** |
| *Female, n (%)* | 224 (40.22) | 144 (63.72) | 79 (58.52) |  |
| *Male, n (%)* | 333 (59.78) | 82 (36.28) | 56 (41.48) |  |
| BMI (kg/m^2^), mean±SD; (95%CI) | 26.20±3.86; (25.92 to 26.49) | 26.26±3.96; (25.80 to 26.71) | 25.64±4.15; (25.07 to 26.20) | 0.15 |
| BMI (kg/m^2^) ≥25, n (%) | 399 (56.68) | 180 (60.40) | 113 (53.81) | 0.14 |
| First-degree family history of DD, n (%) | 129 (18.43) | 65 (21.74) | 60 (28.44) | **0.007** |
| **Lifestyle and dietary factors** |  |  |  |  |
| Active smoking, n (%) | 104 (14.75) | 37 (12.33) | 41 (19.34) | 0.09 |
| Number of cigarettes, mean±SD; (95%CI) | 16.41±11.32; (15.14 to 17.68) | 18.29±15.12; (15.28 to 21.31) | 17.51±12.94; (14.77 to 20.25) | 0.39 |
| Use of coffee, n (%) | 195 (34.88) | 76 (32.20) | 56 (33.73) | 0.76 |
| Units of coffee, mean±SD; (95%CI) | 2.28±1.24; (2.18 to 2.39) | 2.23±1.42; (2.05 to 2.42) | 2.16±1.23; (1.97 to 2.35) | 0.58 |
| Diet rich in fibre, n (%) | 94 (13.33) | 46 (15.33) | 39 (18.40) | 0.18 |
| Consumption meat, n (%) | 378 (53.62) | 157 (52.33) | 116 (4.72) | 0.86 |
| **Drugs** |  |  |  |  |
| NSAIDs, n (%) | 35 (4.96) | 15 (5.00) | 6 (2.83) | 0.33 |
| Antiplatelet agents, n (%) | 156 (22.13) | 63 (21.00) | 40 (18.87) | 0.59 |
| Statin, n (%) | 141 (20.06) | 68 (22.82) | 41 (19.43) | 0.55 |
| Rifaximin, n (%) | 81 (11.49) | 92 (30.67) | 74 (34.91) | **<0.0001** |
| Mesalazine, n (%) | 8 (1.13) | 12 (4.00) | 30 (14.15) | **<0.0001** |
| Prebiotics, n (%) | 4 (0.57) | 6 (2.00) | 4 (1.89) | 0.07 |
| Probiotics, n (%) | 8 (1.13) | 23 (7.67) | 18 (8.49) | **<0.0001** |

*Legend: BMI: body mass index; DD: diverticular disease; NSAIDs: Non-steroideal anti-inflammatory drugs; SUDD: symptomatic uncomplicated diverticular disease*

Post-hoc Analysis:

**Sex:** Female and Male: Diverticulosis vs. SUDD, p<0.0001; Diverticulosis vs. Previous Diverticulitis, p=0.002; SUDD vs. Previous Diverticulitis, p=0.10

**Age**: Diverticulosis vs. SUDD, p=0.53; Diverticulosis vs. Previous Diverticulitis, p=0.0006; SUDD vs. Previous Diverticulitis, p=0.01

**Age classes:** <60 years and ≥60 years: Diverticulosis vs. SUDD, p=0.20; Diverticulosis vs. Previous Diverticulitis, p<0.0001; SUDD vs. Previous Diverticulitis, p=0.004

**Age classes*Sex: ≥60 years:** Female and Male: Diverticulosis vs. SUDD, p<0.0001; Diverticulosis vs. Previous Diverticulitis, p=0.0001; SUDD vs. Previous Diverticulitis, p=0.33

**First-degree family history of DD**: Diverticulosis vs. SUDD, p=0.23; Diverticulosis vs. Previous Diverticulitis, p=0.002; SUDD vs. Previous Diverticulitis, p=0.08

**Use of rifaximin**: Diverticulosis vs. SUDD, p<0.0001; Diverticulosis vs. Previous Diverticulitis, p<0.0001; SUDD vs. Previous Diverticulitis, p=0.31

**Use of mesalazine**: Diverticulosis vs. SUDD, p=0.005; Diverticulosis vs. Previous Diverticulitis, p<0.0001; SUDD vs. Previous Diverticulitis, p<0.0001

**Use of probiotic:** Diverticulosis vs. SUDD, p<0.0001; Diverticulosis vs. Previous Diverticulitis, p<0.0001; SUDD vs. Previous Diverticulitis, p=0.74
