# Supplementary figures and images for: Progression rate of diverticular disease and associated risk factors: results from 5-year longitudinal prospective nationwide diverticular disease registry (REMAD)
Source: Intern Emerg Med. 2026 Feb 26;21(4):1215–25. doi: 10.1007/s11739-026-04283-4 (PMC13263300; doi:10.1007/s11739-026-04283-4)

## Acute diverticulitis incidence in diverticulosis

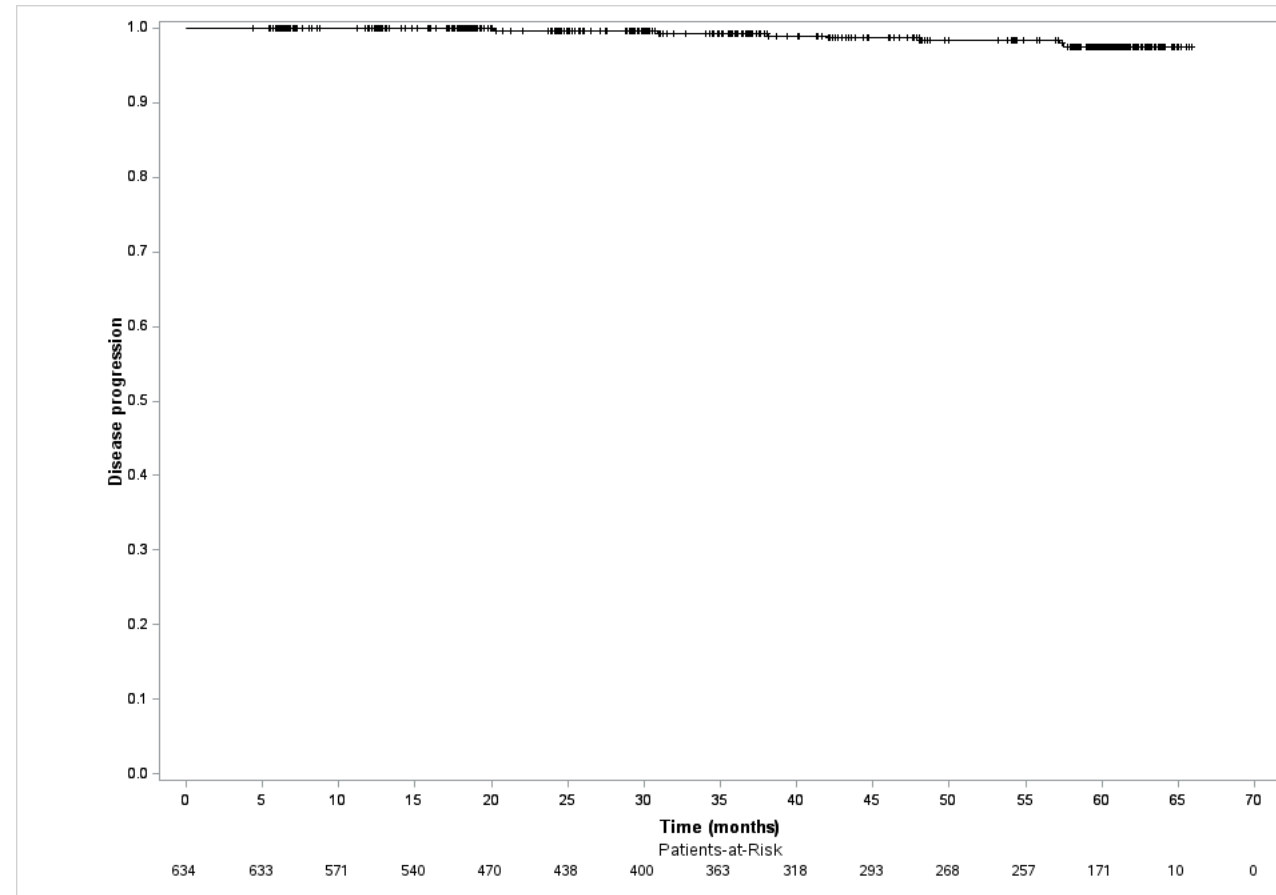

Supplement: Supplementary file 2 — Supplementary file2 (PDF 52 KB) [file 11739_2026_4283_MOESM2_ESM.pdf]

SUDD incidence in diverticulosis

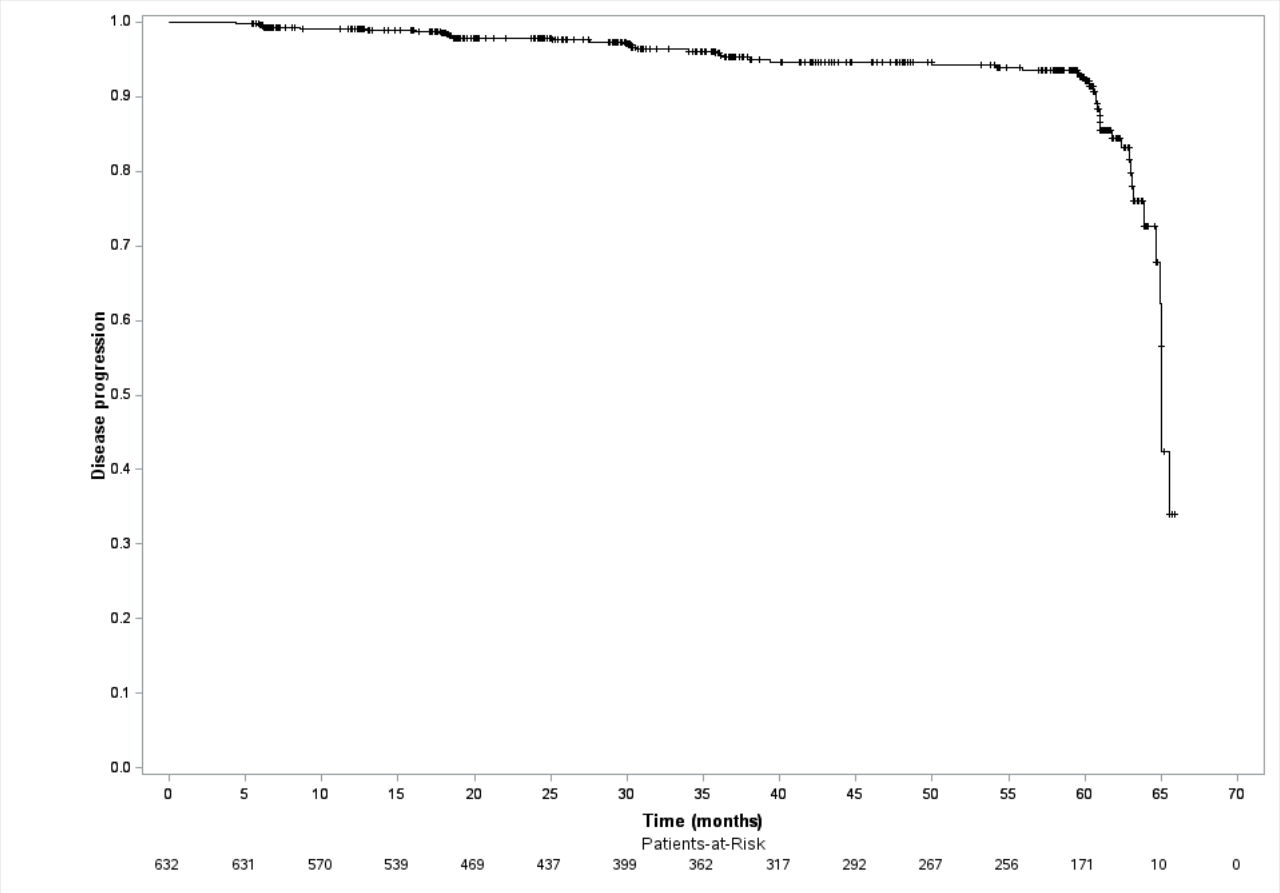

Supplement: Supplementary file 3 — Supplementary file3 (PDF 54 KB) [file 11739_2026_4283_MOESM3_ESM.pdf]

Acute diverticulitis incidence in SUDD

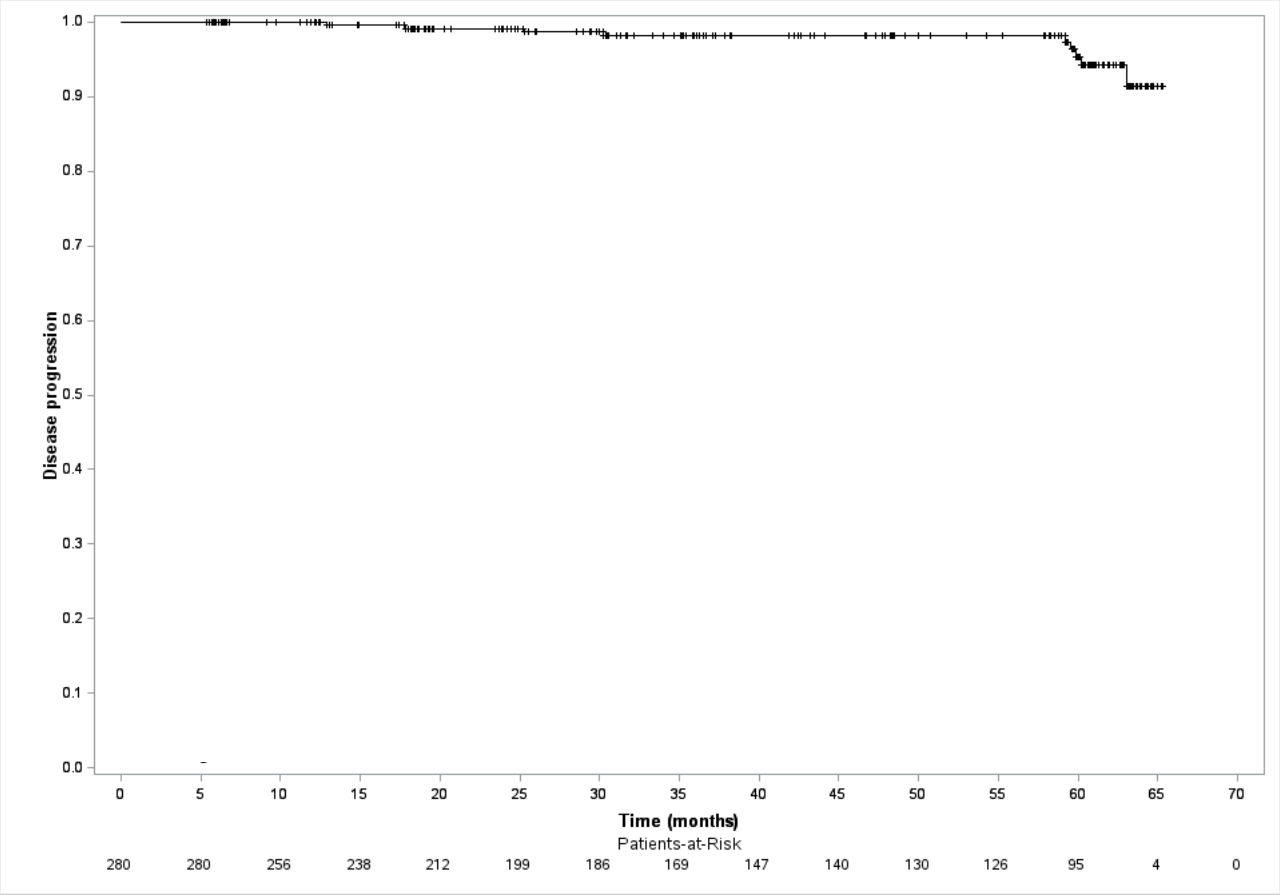

Supplement: Supplementary file 4 — Supplementary file4 (PDF 51 KB) [file 11739_2026_4283_MOESM4_ESM.pdf]

## Recurrent diverticulitis

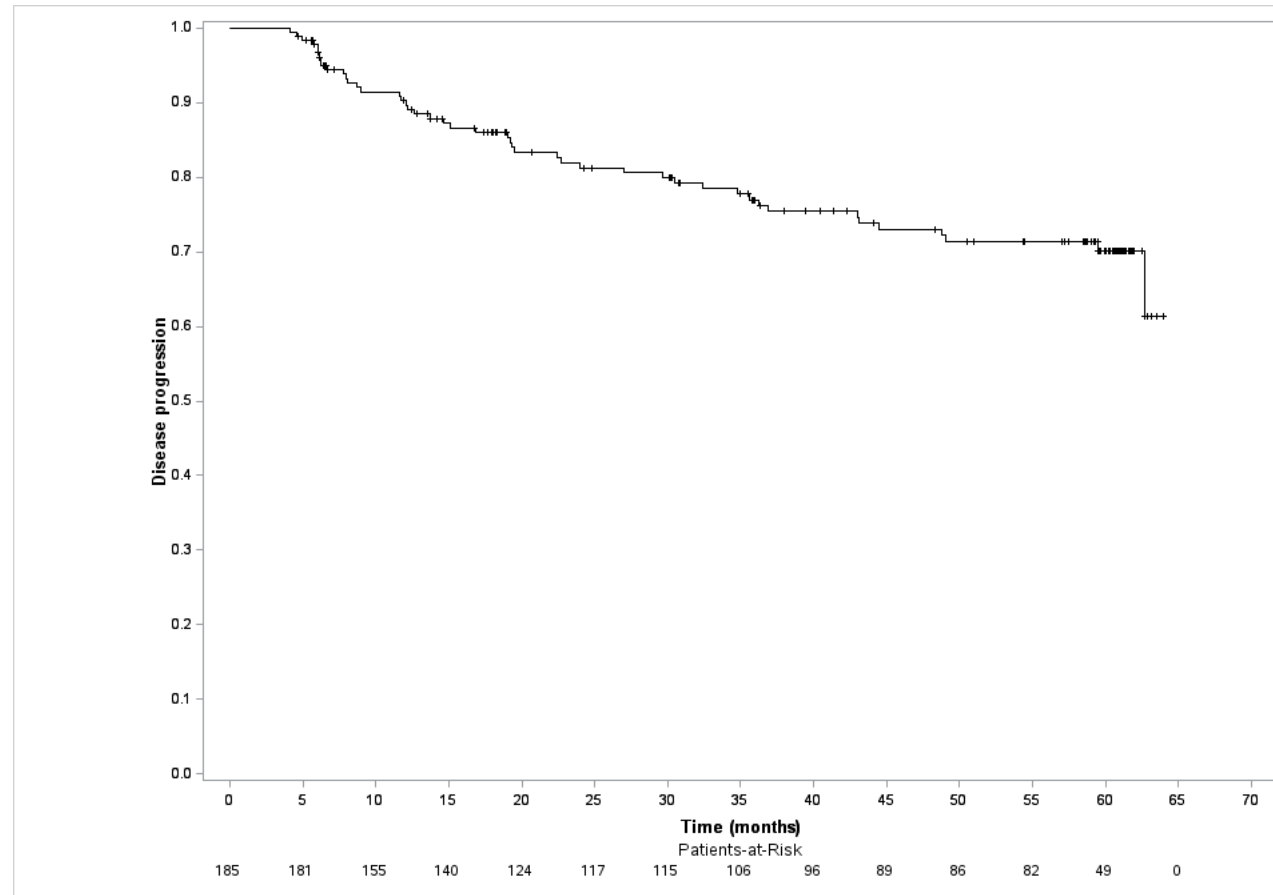

Supplement: Supplementary file 5 — Supplementary file5 (PDF 51 KB) [file 11739_2026_4283_MOESM5_ESM.pdf]
